# Dysbiosis signatures of gut microbiota and the progression of type 2 diabetes: a machine learning approach in a Mexican cohort

**DOI:** 10.3389/fendo.2023.1170459

**Published:** 2023-06-27

**Authors:** Daniel Neri-Rosario, Yoscelina Estrella Martínez-López, Diego A. Esquivel-Hernández, Jean Paul Sánchez-Castañeda, Cristian Padron-Manrique, Aarón Vázquez-Jiménez, David Giron-Villalobos, Osbaldo Resendis-Antonio

**Affiliations:** ^1^ Human Systems Biology Laboratory, Instituto Nacional de Medicina Genómica (INMEGEN), México City, Mexico; ^2^ Programa de Maestría y Doctorado en Ciencias Bioquímicas, Universidad Nacional Autónoma de México (UNAM), Ciudad de México, Mexico; ^3^ Programa de Doctorado en Ciencias Biomédicas, Universidad Nacional Autónoma de México (UNAM), Ciudad de México, Mexico; ^4^ Coordinación de la Investigación Científica – Red de Apoyo a la Investigación, Universidad Nacional Autónoma de México (UNAM), Ciudad de México, Mexico; ^5^ Centro de Ciencias de la Complejidad, Universidad Nacional Autónoma de México (UNAM), Ciudad de México, Mexico

**Keywords:** type 2 diabetes, Mexican patients, microbiota, machine learning, explainable artificial intelligence, dysbiosis, SHAP value

## Abstract

**Introduction:**

The gut microbiota (GM) dysbiosis is one of the causal factors for the progression of different chronic metabolic diseases, including type 2 diabetes mellitus (T2D). Understanding the basis that laid this association may lead to developing new therapeutic strategies for preventing and treating T2D, such as probiotics, prebiotics, and fecal microbiota transplants. It may also help identify potential early detection biomarkers and develop personalized interventions based on an individual’s gut microbiota profile. Here, we explore how supervised Machine Learning (ML) methods help to distinguish taxa for individuals with prediabetes (prediabetes) or T2D.

**Methods:**

To this aim, we analyzed the GM profile (16s rRNA gene sequencing) in a cohort of 410 Mexican naïve patients stratified into normoglycemic, prediabetes, and T2D individuals. Then, we compared six different ML algorithms and found that Random Forest had the highest predictive performance in classifying T2D and prediabetes patients versus controls.

**Results:**

We identified a set of taxa for predicting patients with T2D compared to normoglycemic individuals, including *Allisonella, Slackia, Ruminococus_2, Megaspgaera, Escherichia/Shigella, and Prevotella*, among them. Besides, we concluded that *Anaerostipes, Intestinibacter, Prevotella_9, Blautia, Granulicatella*, and *Veillonella* were the relevant genus in patients with prediabetes compared to normoglycemic subjects.

**Discussion:**

These findings allow us to postulate that GM is a distinctive signature in prediabetes and T2D patients during the development and progression of the disease. Our study highlights the role of GM and opens a window toward the rational design of new preventive and personalized strategies against the control of this disease.

## 1 Introduction

The study of host-microbiota associations has opened a window of opportunities for detecting the progression of complex human diseases and designing new treatments and preventive strategies (1). Notably, promising results have been found in association models between the composition of the human gut microbiota (GM) and the individual phenotype, specifically for complex diseases such as colorectal cancer, inflammatory bowel disease, liver cirrhosis, and type 2 diabetes (T2D) (2). A direct relation between individuals with T2D and dysbiosis of GM has been described during the progression of the disease (3). Furthermore, this is associated with an increase in gut permeability, low-grade systemic inflammation, and inadequate modulation of the immune system and glucose metabolism by the metabolites derived from the GM, including short-chain fatty acids (SCFAs) and secondary bile acids (BAs) in the human body.

Therefore, some efforts have been made to identify this association to develop individualized diagnostic and therapeutic interventions in patients with T2D or prediabetes, with a particular focus on developing countries, given the high mortality and prevalence in these populations (4). In addition, the association between GM and T2D varies depending on geographic variables. For example, a decrease in butyrate-producing species, such as *Roseburia intestinalis* and *Faecalibacterium prausnitzii*, was described in Chinese patients with T2D. In contrast, a second study in European patients with T2D found dysbiosis in certain species, such as *Lactobacillus gasseri, Streptococcus mutans*, and *Clostridium clostridioforme* (5). In this research, they found that these three species were increased in patients with T2D, and several of them were linked to other clinical variables. For instance, the levels of triglycerides and C-peptide were positively associated with *Clostridium clostridioforme*, while fasting glucose and HbA1c were strongly correlated with *Lactobacillus gasseri* (5). It’s also critical to remember that several of these species are opportunistic pathogens, including *Clostridium clostridioforme* and *Streptococcus mutans*, which have been connected to bacteremia and human infections (6). These results suggest that the gut microbiota’s composition, including the prevalence of unique bacterial species, may have a major influence on the onset and course of T2D.

The study of the relationship between microbiota and T2D is challenging due to several factors. Firstly, the human microbiota is highly diverse, and its composition and function vary significantly between individuals. Secondly, T2D is a multifactorial and heterogeneous disease involving complex interactions between genetic, environmental, and lifestyle factors. Thirdly, microbiota data analysis requires advanced computation and statistical methods to handle high-dimensional, sparse, and noisy data. For this reason, researchers proposed several supervised Machine Learning (ML) methods in combination with *post hoc* explanation approaches to improve classification predictions and identify the microbiome-disease association simultaneously (7). In addition, recent advances in artificial neural networks (ANNs) have attracted attention due to their high predictive ability. ANNs are powerful ML techniques used to extract and transform information using multiple layers of neural networks. These layers receive information from previous layers and are progressively refined to improve prediction accuracy. ANNs are known for their high predictive ability but can be prone to overfitting and require large amounts of training data (8).

On the other hand, the use of tree-based ML methods, such as XGBoost and Random Forest, on microbiome data has obtained comparable performance to ANNs and may handles better small datasets (9). XGBoost is a tree-based ensemble learning method that uses a set of uncorrelated decision trees depending upon several randomly selected variables. It iteratively creates new decision trees by calculating the error of the previous model until the highest prediction is found. Similarly, Random Forest is also a tree-based ensemble learning method that creates a set of decision trees by randomly selecting a subset of features at each node to reduce overfitting. Compared to ANNs, tree ensemble models such as XGBoost are more suitable for small sample size and class imbalance datasets than different ANNs architectures for tabular data (10).

We suggest different classification ML methods to predict the clinical phenotypes of naive Mexican patients with T2D or prediabetes. Thus, we compared the performance of six different ML algorithms (see methods) to classify the individual state of health vs. disease status using the GM data characterized by 16s rRNA gene metabarcoding.

Following this, we proposed to select the model with the best predictive performance for an explanatory model analysis using a *post-hoc* algorithm called SHapley Additive exPlanations (SHAP) (11). Using the SHAP values, we try to identify the specific bacterial taxa that played a crucial role in classifying health versus disease status. Furthermore, this approach may provide a comprehensive, model-agnostic, and interpretable explanation of the ML model’s predictions (11). Our findings could provide valuable insights into the underlying mechanisms linking the GM to developing T2D and prediabetes.

## 2 Results

In our study, we compared different ML methods to determine the most effective approach for classifying individuals with prediabetes or Type 2 Diabetes (T2D) compared to the control group. Since each ML method has different characteristics, evaluating several algorithms to find the best fit for our cohort was essential (**supplementary Table 1**). The model with the best overall performance was analyzed using the SHAP values to find the most critical taxa to distinguish the groups.

**Figure 1:**
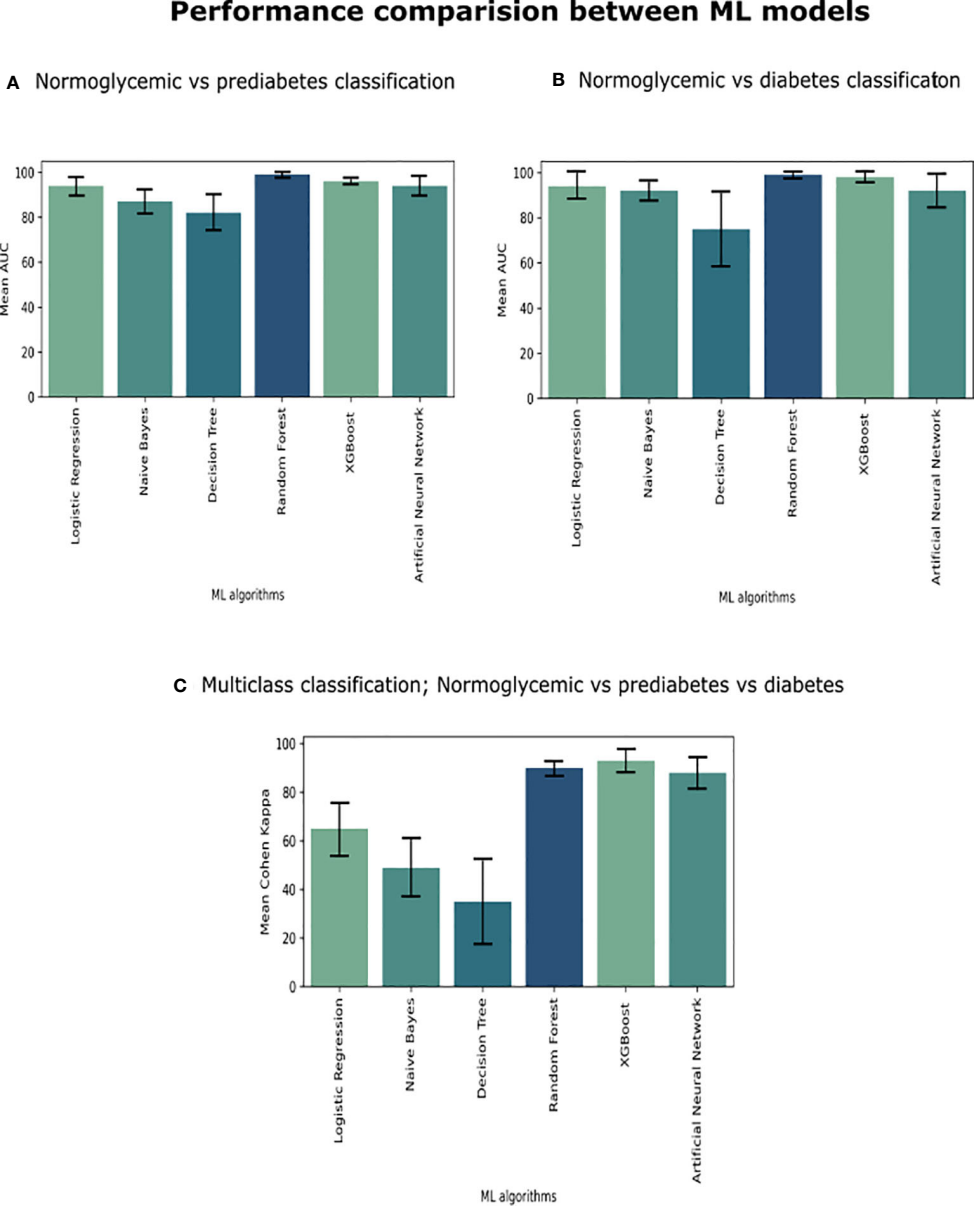
We compared six ML algorithms in three classifications **(A)** Individuals with NGT vs. Patients with prediabetes, **(B)** Patients with NGT vs. Patients with T2D, and **(C)** Patients with NGT vs. Patients with prediabetes vs. Patients with T2D. We plot a standard error bar of the area under the receiver operating characteristic (ROC) curve (AUC) median values for a visual comparison performance between the models in each classification. In the case of multi-class classification (part C), we evaluated it using Cohen Kappa Score.

To achieve this, we performed three comparisons: classification 1 (C-1) compared individuals with NGT versus patients with prediabetes; classification 2 (C-2) compared individuals with NGT versus patients with T2D; classification 3 (C-3) we performed a multi-class classification to predict individuals with NGT, prediabetes, and T2D. For each classification, we evaluated the predictive performance of six different algorithms: Binary Logistic Regression, Naive Bayes, Decision Tree, Random Forest, XGBoost, and Multilayer Perceptron (MLP) (**Figure 1**).

### 2.1 Classification (C1): NGT versus prediabetes

The models in C1 with the best predictive values were Random Forest (mean accuracy= 0.98, standard deviation (SD) 0.02) and MLP (mean accuracy= 0.94, SD 0.02). The best models based on the AUC-ROC metric were Random Forest (mean AUC = 0.99, SD 0.01), followed by MLP (mean AUC= 0.94, SD 0.03) (**Figure 1A**).

**Figure 2:**
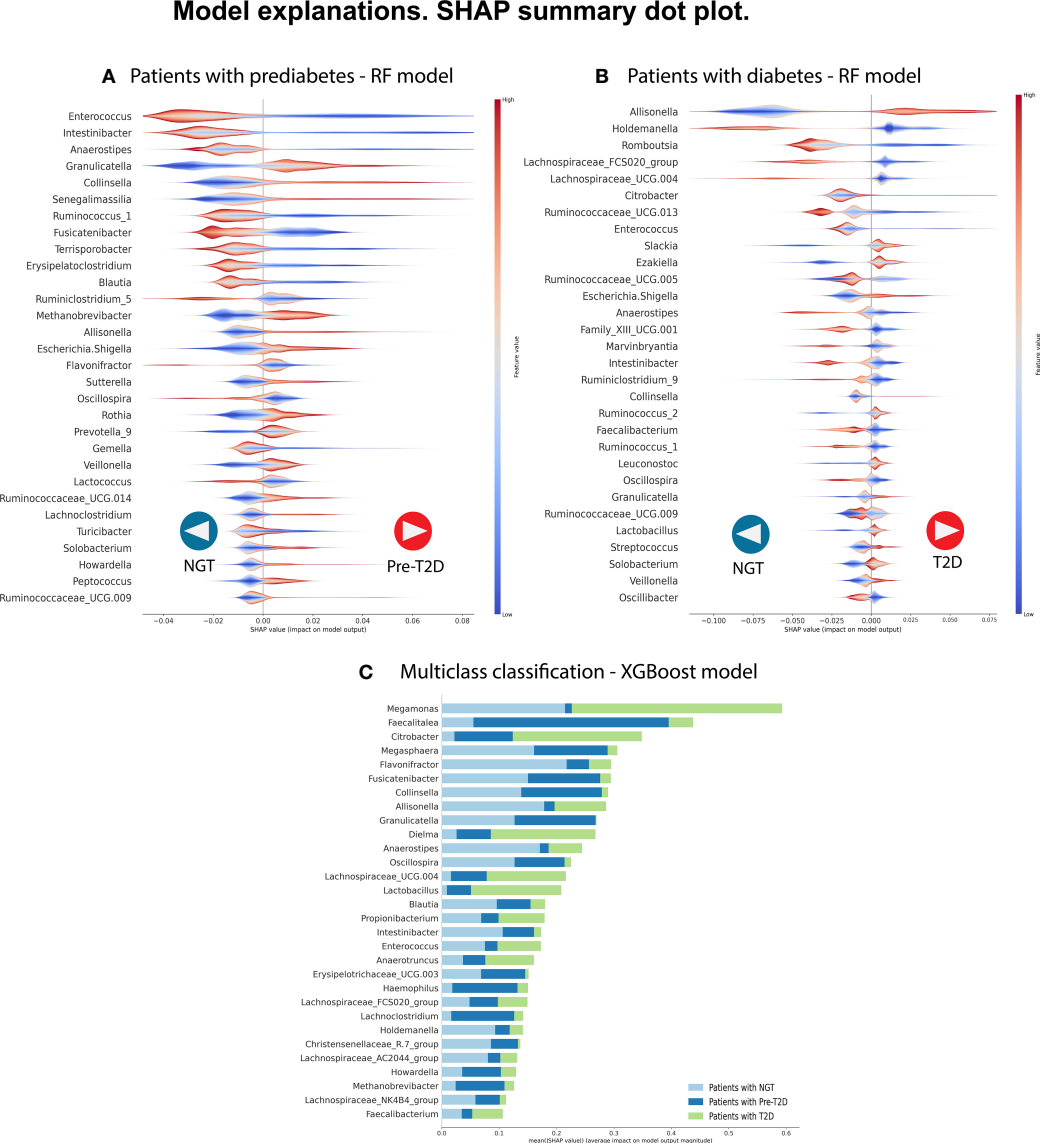
We selected the best predictive model in each classification to identify the most relevant bacterial genera to classify the phenotype. We analyze the three comparisons. **(A)** Patients with NGT (n= 213) versus patients with prediabetes (n= 47). **(B)** Patients with NGT (n= 213) versus patients with T2D (n= 47). **(C)** Multi-class classification: Patients with NGT vs. patients with prediabetes (n=150) vs. patients with T2D (n= 47). In each, a graph of the SHAP values shows, in hierarchical order, the 30 most important bacterial genera and their type of influence on the Random Forest model (in parts A and B) and the XGBoost model (in part C). In parts A and B, positive SHAP values help predict individuals with disease (prediabetes or T2D), whereas negative SHAP values help predict individuals with NGT. The color code shows the feature values, in this case representing the relative abundance of the bacterial genera, with red color showing high relative abundance values and blue color showing low relative abundance values in the samples. In part, C shows the most important bacterial genera with their mean SHAP value and uses a color code to indicate the label of the predicted class.

Therefore, the Random Forest model was analyzed using the SHAP values to show the most important genera to identify the groups and their influence on the output (**Figure 2A**). Some of the most important bacterial genera for classification found were *Intestinibacter, Anaerostipes, Enterococcus, Collinsella, Fusicatenibacter*, and *Granulicatella.* Low relative abundance values of *Intestibacter, Enterococcus*, and *Anaerostipes* help predict patients with prediabetes. And high levels of relative abundance of the genera *Collinsella, Allisonella, Escherichia/Shigella*, and *Senegalimassilia* help to select patients with prediabetes. However, we considered that it is difficult to select a unique taxon to identify individuals with the disease accurately based and can vary depending on the specific dataset or algorithm used. Instead, a more meaningful approach is to identify a set of specific patterns and changes in the complete GM profile of individuals with the disease. This will allow more accurate identification of individuals suffering from the disease.

Random Forest is an ensemble method (combination of multiple classifiers) based on generating a set of uncorrelated decision trees to make a prediction, making it robust and suitable for complex data patterns. The model results in this classification allow us to select Random Forest as the best method for this task because of its ability to capture nonlinear interactions in tabular data. Other studies have found that Random Forest’s performance is on par with deep learning algorithms when applied to several microbiome sets from different populations (7). These findings highlight the strength of Random Forest as a machine-learning method for classification tasks in microbiota data.

### 2.2 Classification C2: NGT versus T2D

The models with the best accuracy values in C2 were also Random Forest (mean accuracy= 0.96, SD 0.03), followed by MLP (mean accuracy= 0.91, SD 0.07), and XGBoost (mean accuracy= 0.91, SD 0.07). The models with better AUC values in C2 were Random Forest (mean AUC = 0.99, SD 0.01) and MLP (mean AUC= 0.98, SD 0.02) (**Figure 1B**).

The Random Forest model demonstrated the highest predictive performance in classifying individuals with T2D in the C2 model. For this reason, we analyzed this model using the SHAP values to identify the most important bacterial genera useful for predicting NG individuals or individuals with T2D. **Figure 2B** shows the top 30 of bacterial genera most responsible for the model output in the order of importance. High relative abundance levels of *Escherichia/Shigella, Slackia*, and *Allisonella* help select patients with T2D. And high levels of relative abundance of *Lachnospiracea_UCG.004, Holdemanella*, *Ruminococcus_1*, and *Anaerostipes* help to predict individuals with NGT. For some taxa, we did not see a specific pattern of high or low abundance values of the specific genre to classify individuals with T2D or the control group.

### 2.3 C3: Individuals with NGT vs. prediabetes vs. T2D

The models in C3 with the best predictive values were XGBoost (Mean Accuracy= 0.96, SD 0.02) and Random Forest (Mean Accuracy= 0.95, SD 0.03). According to the Cohen Kappa metric, the best models were XGBoost (Mean Cohen Kappa score = 0.93, SD 0.05) followed by Multinomial Logistic Regression (Mean Cohen Kappa score= 0.94, SD 0.03) (**Figure 1C**). ​​We chose to use Cohen’s Kappa over the AUC, because it can be difficult to interpret in multi-class classification using AUC, as it requires converting the problem into a set of binary classification tasks. On the other hand, Cohen’s Kappa offers a single score that accounts for the agreement between the predicted and actual labels for every class, making it a better measurement for our multi-class classification task.

The XGBoost model of C3 (multiclass) demonstrated the highest predictive performance in classifying individuals with NGT, prediabetes, or T2D. We analyzed this model using the SHAP values to identify the major bacterial genera for multilabel classification of individuals with NGT vs. individuals with prediabetes vs. individuals with T2D.

**Figure 2C** displays the 30 most influential bacterial genera, ranked in order of importance. Some genera include *Megamonas, Faecalitalea, Citrobacter, Megasphera Intestinibacter, Anaerostipes, Allisonella, Collinsella, Fusicatenibacter, Dielma, Oscillospiram, Blautia*, and *Granulicatella.*

## 3 Discussion

GM has been an emerging factor in the pathogenesis of T2D, related to the patient´s environmental risks factors such as diet, obesity, sedentary lifestyles, and genetic risk factors, including specific genetic variants (12). However, studying the relationship between the host and GM is complex, and identifying the possible keystone taxa associated with the prediabetic or diabetic stage is still problematic (13).

To address this issue, we evaluated various supervised ML methods to identify specific patterns and alterations in the GM profiles of patients with prediabetes and T2D. Tree-based algorithms such as Random Forest and XGBoost provided the best predictive performance in our cohort. Furthermore, the *post-hoc* analysis of the models enabled us to understand the impact of keystone taxa on patients with T2D or prediabetes compared with negative control. The most critical genera identified with these models were *Escherichia/Shigella*, *Anaerostipes, Blautia*, *Roseburia*, and *Collinsella*. Likewise, some studies describe these keystone taxa as having a role in the pathogenesis of T2D in different populations (3, 14).

In addition to the typical methods used to study the microbiome, using Deep Learning algorithms such as MLP is an attractive alternative to finding robust and high predictive performance results (15). However, using them on small datasets that commonly suffer in these microbiome studies remains challenging, which makes the models easily susceptible to *overfitting* (16). We believe that new approaches will continue to be developed to improve the analysis of small datasets. Using a larger amount of data to train the deep learning model would allow finding better performance results reaching the potential reported in other studies. With the results in our cohort, we can conclude that the methods based on decision trees (Random Forest, XGBoost) allow a better understanding of the model and better performance than the deep learning models in our case.

**Figure 3:**
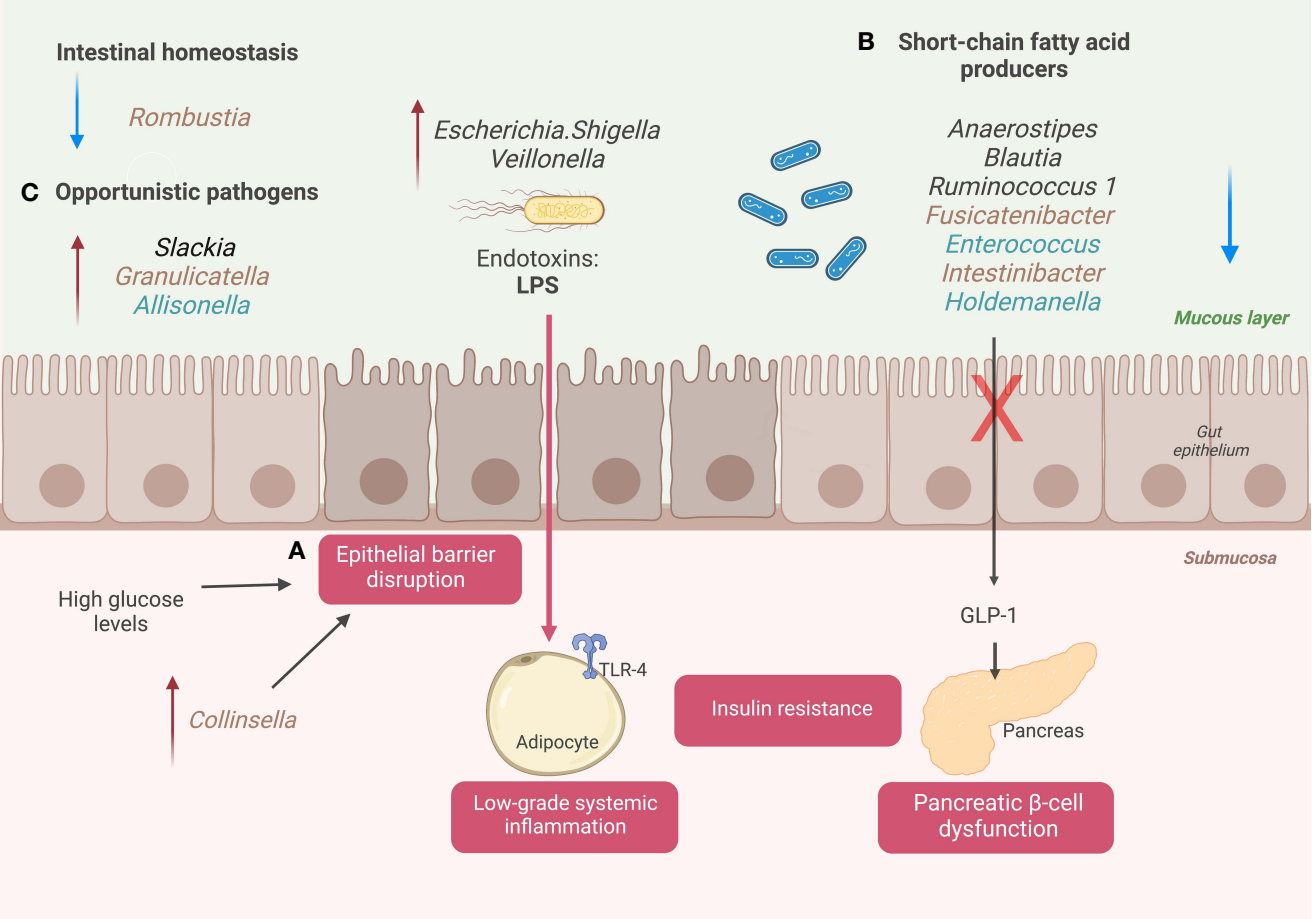
A schematic diagram illustrates the changes in GM associated with Mexican patients with T2D. **(A)** Disruption of the epithelial gut barrier and metabolic endotoxemia. **(B)** SCFA’s producer’s genera and pancreatic beta cell dysfunction. **(C)** Alter gut homeostasis and increase opportunistic taxa. Genera colored with brown for prediabetic patients and genera colored with blue for diabetic patients. Created with BioRender.com.

Furthermore, we explore the possible keystone taxa we found with Tree-based ensemble learning methods, including Random Forest and XGBoost. Here we summarize the changes in the structure of the GM and their association with the disease progression in a cohort of Mexican patients with T2D or prediabetes: 1) increase intestinal permeability and metabolic endotoxemia, 2) reduction of SCFAs genera producers, 3) alteration gut homeostasis and increase opportunistic pathogens. (**Figure 3**)

### 3.1 Increase gut permeability and metabolic endotoxemia

High blood glucose levels are associated with a loss of gut epithelial integrity, driving the passage of endotoxins (lipopolysaccharides (LPS)) and other microbial components into the bloodstream (17). This phenomenon is called metabolic endotoxemia and can trigger the systemic immune response (18). The interaction between microbial components and innate immune receptors activates the expression of proinflammatory cytokines (interleukin-4 (IL4), interleukin-6 (IL6), and tumor necrosis factor-α (TNF-α)) in different insulin sensitive-tissues and blood vessels (**Figure 3**). The increase of these mediators maintains a chronic low-grade systemic inflammation state associated with T2D progression and long-term vascular complications (19).

During our study, we detected relevant genera associated with the loss of gut epithelial integrity and metabolic endotoxemia that helped to classify diabetic or prediabetic patients compared to NGT subjects. We found that high levels of relative abundance in *Escherichia/Shigella* and *Veillonella*, gram-negative genera with LPS in their walls, helped predict patients with prediabetes or with T2D when compared with NGT subjects. *Escherichia/Shigella* was on the top 30 essential taxa to classify prediabetes (C2: NGT vs. prediabetes) and T2D (C1: NGT vs. T2D) (**Figure 2A, B**). In addition, *Veillonella* was also on the top 30 key taxa to classify patients with T2D (**Figure 2B**). According to the literature, these LPS-producing genera, *Escherichia/Shigella*, and *Veionella*, have high abundance levels in patients with T2D in different populations (20–22). In addition, these genera have been related to other pathologies with intestinal dysbiosis as a common component in their pathophysiology, for example, irritable bowel syndrome and inflammatory bowel disease.

In patients with T2D, the increase in gut permeability could be attributed to other factors such as long-term consumption of a processed diet, drugs, alcohol consumption, and gut dysbiosis (23). For this, specific taxa in the GM may directly affect epithelial integrity. Some studies point to *Collinsella* having a particular role in this phenomenon. *Collinsella* disrupts the intestinal barrier by decreasing the expression of thigh junction proteins in enterocytes (24). We found that an increase in the abundance levels of *Collinsella* help classifies individuals with prediabetes in the top 30 ranked genera. (C2: NGT vs. prediabetes) (25).

Metabolic endotoxemia due to gut dysbiosis is a component in patients with diabetes that perpetuates the chronic inflammatory state and disease progression. Currently, there are no interventions with the immediate objective of restoring epithelial integrity. However, a set of treatments (e.g., fecal microbiota transplantation, diet, drugs, and prebiotics) considering the microbiota could help to reduce systemic inflammation and its complications caused by increased intestinal permeability in these patients (26). For instance, prebiotics such as fructooligosaccharides (FOS) and inulin can promote the development of beneficial gut bacteria and improve the efficiency of the gut barrier. It has also been demonstrated that probiotics, such as strains of *Lactobacillus* and *Bifidobacterium*, enhance gut barrier function and lessen inflammation. Whereas they present exciting opportunities for future therapeutic interventions, further study is required to fully understand the possible advantages and dangers of these treatments for people with T2D or prediabetes.

### 3.2 SCFAs producers and beta cell dysfunction

Concerning dysbiotic microbiota, patients with T2D have a decrease in certain bacterial producers of SCFAs, including butyrate, propionate, and acetate. In addition, some environmental factors in diabetic patients, such as a Western-type diet (low in fiber, rich in calories from saturated fatty acids and sugars), are associated with a decrease in butyrate-producing species (e.g., *Roseburia intestinalis* and *Faecalibacterium prausnitzii*) (27, 28). The low production of SCFAs is associated with alterations in insulin sensitivity and inadequate immune system modulation, which are risk factors for prediabetes and T2D (29). We have found that low relative abundance levels of SCFAs producer’s genera, such as *Anaerostipes, Enterococcus, Intestinibacter*, and *Fusicatenibacter*, help to classify patients with T2D and patients with prediabetes compared with NGT patients. *Anaerostipes* was the third most crucial variable out of 150 genera studied for classifying patients with prediabetes (C1: NGT vs. prediabetes), and the 12° most important bacterial genera for classifying patients with T2D (C2: NGT vs. T2D). In addition to their role in insulin sensitivity modulation, SCFAs function to maintain a typical phenotype of colonocytes in the human intestine, providing survival and anti-apoptosis signals (29). A healthy gut epithelium prevents the passage of microorganisms and their subsequent activation of the immune system in an altered way (30).

These SCFAs metabolites act through G protein-coupled receptors (including GPR41, GPR43, and GPR109A), expressed in several tissues: intestinal epithelial cells, adipose tissue, and immune cells. For this reason, they have pleiotropic functions related to the digestive, immune, and neuroendocrine systems (31). For example, SCFAs stimulated the secretion of satiety-related peptides (peptide YY and leptin) and modulated the function of macrophages, dendritic, and T and B cells. Together, these functions help to maintain local and overall homeostasis in the human body (32).

Therefore, it is essential to measure the luminal metabolites associated with these mechanisms in patients with T2D or prediabetes to discover new insights and approaches to prevent the disease progression.

### 3.3 Alter gut homeostasis and increase opportunistic genera

One main change in the gut microbiome composition in patients with T2D is the increase of opportunistic pathogens accompanied by a decrease in SCFAs-producing genera (6). In a Chinese cohort, they shown are an increase in several opportunistic pathogens, including *Escherichia coli*, *Bacteroides caccae, Clostridium hathewayi, Clostridium ramosum, Clostridium symbiosum*, and *Eggerthella lenta (* 33 *).* In other cohorts, describe a change in bacterial species associated with intestinal health. For example, *L. acidophilus* or *L. salivarius*, but some species, such as *L. amylovorus*, are negatively associated with diabetes.

In our cohort, *Collinsella* and *Lachnoclostridium* are among the top 30 bacterial genera, being useful for classifying individuals with prediabetes (C1: NGT vs. prediabetes). These bacterial genera are associated with high levels of Trimethylamine (TMA), a pro-inflammatory metabolite associated with vascular complications (34). TMA is produced by the GM from L-carnitine, choline, and betaine in high amounts in red meat and fatty foods. In the liver, TMA is converted to TMAO (oxidized TMA) by the enzyme FMO3 (flavin 3-containing monooxygenase) (35). High levels of TMAO play a critical role in the formation of atherosclerosis. TMAO induces an inflammatory response at the vascular level, causing endothelial dysfunction and altering cholesterol metabolism (34, 36). Moreover, TMAO could be related as a determinant factor in the mortality of these patients. Thus, subjects with T2D and prediabetes have an increased risk of developing cardiovascular disease (CVD) (37, 38).

Furthermore, we performed the Linear Discriminant Analysis Effect Size (LEfSe) method to identify particular differences in the bacterial phenotype at the genus level between the normoglycemic, pre-T2D, and T2D groups (**Supplementary Figure 6** and **Supplementary Figure 7**) (39). Our findings revealed distinct microbial signatures associated with each group. Regarding the T2D group, several taxa, including *Enterobacterales*, *Enterobacteriaceae*, *Escherichia/Shigella*, *Gammaproeteobacteria*, *proteobacteria*, *Fusicatenibacter*, *Lactobacillus*, *Dielma*, and *Allisonella*, were significantly enriched, pointing to a dysbiotic pattern. In the prediabetes group, we found the following taxa with substantial changes, including *Selenomonadales, Negativicutes, Megasphera, Methanobacteria, Veillonellaceae, Howardella*, and *Butyrimonas*. Interestingly, the normoglycemic group showed a unique pattern, with taxa like *Clostridia, Clostridiales, Firmicutes, Lachnospiraceae, Blautia, Anaerostipes*, and *Rombustia*. These results highlight the potential of the gut microbiota as a biomarker for the development of T2D and shed light on bacterial taxa that might play a role in disease pathogenesis.

Overall, using the LEfSe method, we identified microbial signatures linked to various prediabetic and diabetic stages and highlighted particular taxa that may potentially contribute to the onset and progression of T2D. Some of them were also identified as important to distinguish between groups using ML models, including *Escherichia/Shigella, Allisonella, Dielma, Howardella, Blautia, Anaerostipes, Rombustia*, and *Lactobacillus.* We can recognize microbial species whose abundance considerably varies between several groups using LEfSe analysis. To fully understand the intricate connection between gut microbiota and metabolic health, we proposed to use ML explainable analysis to identify the influence in the classification result. The relative significance of each microbiological genus in impacting the ML model’s decision-making can be explained by the SHAP values. They give us the ability to determine which bacterial genera have the greatest influence on the categorization result and to comprehend the underlying mechanisms causing the disease to progress.

In general, this highlights the importance of medical ecology as a useful approach to understanding human health and disease through the lens of the environment, including factors such as diet, lifestyle, and the microbiome. In this context, disease progression in patients with T2D could reflect the dynamic changes exhibited by the GM. Therefore, understanding their ecological associations could allow intervention in the natural history of the disease with personalized interventions, such as individualized nutritional therapies. However, claiming that a single variable or a single taxon is useful for classifying healthy or diseased patients is difficult because GM is a complex system (3). A broad characterization of the GM profile or a group of microbial taxa is necessary to find optimal values for the predictive performance of models due to the complexity and heterogeneity of individuals with T2D (14, 22).

To better understand the implications of our results, shotgun sequencing is an attractive methodology that could characterize the GM at strain or species levels and allow us to study the metabolic capabilities of the GM (3). Additionally, using metabolomics GM data from T2D patients may help to understand the specific implications of the disease progression. Some metabolites of interest include SCFAs, secondary BAs, branched-chain amino acids, indole-derived amino acids, and TMAO (40).

In summary, the work developed in this paper allows us to uncover a unique GM structure in the different T2D stages. We consider intestinal dysbiosis not only a reflection of the pathological state of the individual but also actively participates in favoring the progression of the disease. Modulating the GM through personalized interventions, such as prebiotics, probiotics, or fecal microbiota transplantation, may help to restore intestinal homeostasis and improve metabolic health in T2D and prediabetic patients.

## 4 Methods

As part of a previous study conducted by our laboratory group (22), a total of 410 Mexican individuals without prior diagnosis or treatment were stratified into individuals with normal glucose tolerance (NGT) (n= 213), patients with prediabetes (n= 150), and patients with T2D (n= 47). Patients were classified as prediabetic if they had fasting plasma glucose levels of 100-125 mg/dl (known as impaired fasting glucose (IFG)) and/or 2-hour plasma glucose of 140-199 mg/dl (known as impaired glucose tolerance (IGT)). Patients with a fasting glucose level of > 126 mg/dl and/or a 2-hour plasma glucose level of > 200 mg/dl were classified as T2D.

### 4.1 Intestinal microbiome - 16s rRNA gene sequencing

**Figure 4:**
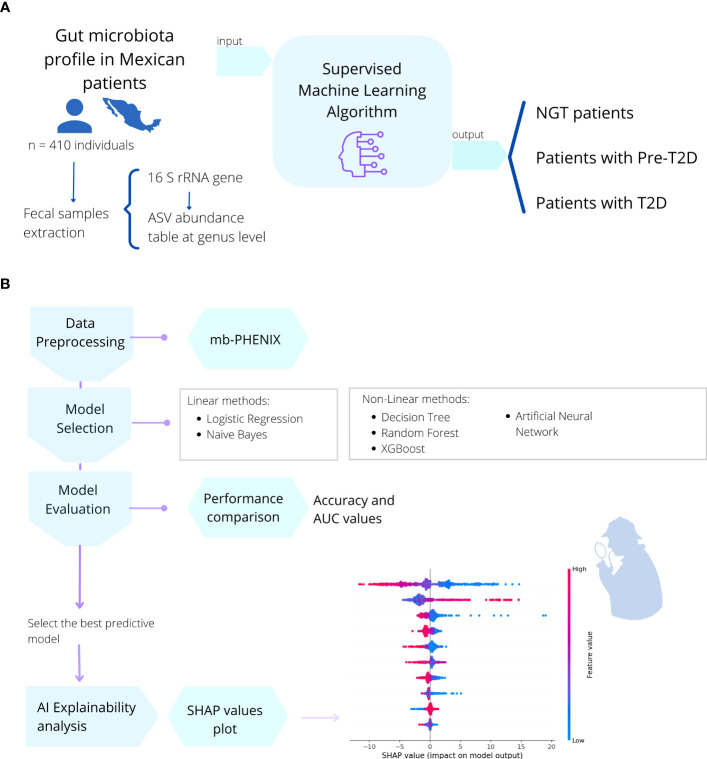
Description of the methods used in this work. **(A)** In a cohort of 410 Mexican patients with different stages of T2DM (prediabetes and T2D) and individuals with NGT, the gut microbial profile (16s rRNA) was obtained from fecal samples. **(B)** Then, six different machine learning algorithms (Binary Logistic Regression, Naive Bayes, Decision Tree, Random Forest, XGBoost, Multilayer Perceptron) were applied to train the model and predict the health or disease status of the individual. We selected the best predictive model and performed a *post-hoc* method interpretation with SHAP values to find the most important gut microbial genera to correctly classify individuals with NGT, prediabetes, or T2D patients,.

To obtain the gut microbiome profile, we used processed sequencing data from Diener et al. https://github.com/resendislab/mext2d. Briefly, as explained by the authors, DNA extraction from the fecal samples and 16 rRNA gene V4 amplicon sequencing was performed. Then, a table of amplicon sequence variants (ASV) was generated using the DADA2 workflow (41), and the taxonomic assignment was performed using the SILVA database v132 (42). This ASV table (analogous to the OTU table), which represents the gut microbiome profiles of each individual, constitutes our starting point of the present work. Following this, we used an artificial intelligence approach using supervised ML methods to create a model capable of understanding the microbiome-disease association (**Figure 4**).

### 4.2 Data pre-processing

In the base pipeline workflow shown in **Figure 4**; the data were first preprocessed using the mb-PHENIX algorithm for each classification independently (13, 43). This was done to address the main problems in microbiota data analysis: first, the sparsity of data with an excess of zeros in the matrix, and second, a lot of features that exceed the observations (high dimensionality). Thus, these issues do not lead to finding data structures using unsupervised dimensionality reduction approaches (13). Taken together, the data nature and the heterogeneity of the phenotype of individuals with diabetes make it difficult overall to identify a unique signature in microbiota data for a specific stage of the disease.

It should be noted that we initially attempted to analyze the data without imputation but ran into problems due to the aforementioned issues. As shown in **Supplementary Figure 1**, the performance of machine learning models was significantly worse without mb-PHENIX preprocessing. Interestingly, as seen in **Supplementary Figure 2**, we also evaluated the best model using summary SHAP plots without mb-PHENIX but found that the interpretability of the model was greatly reduced due to the sparsity and high-dimensionality of the data.

The mb-PHENIX algorithm recovers abundance *via* diffusion based on a supervised UMAP space of the sparse ASV matrix. The initial step from the ASV matrix is to reduce the dimensionality in a supervised manner with UMAP. In brief, the supervised UMAP method aims to map different classes in the low dimensional space as far apart as possible while simultaneously maintaining the internal class structure and the inter-class relationships. Then, this embedding is used for the computation of the Markovian matrix. After, a diffusion process (exponentiation) is applied to the Markovian matrix to refine local neighbors’ similarities. Finally, imputation occurs when the refined (exponentiated) Markovian and ASV matrices are multiplied. Because of that, the missing taxa information is recovered by the local neighbors on the refined Markovian matrix. The construction of the Markovian matrix and the diffusion process of mb-PHENIX is similar to the one from (43); the only difference is that mb-PHENIX uses a supervised UMAP embedding (13). We observed here that mb-PHENIX algorithm can improve the interpretability of the models by making it easier to identify the most important features for predicting the outcome of interest (**Figures 1** and **2**).

We created three imputed matrices with sc-PHENIX based on the following class label information: 1) NGT vs patients with prediabetes, 2) NGT vs patients with T2D, and 3) NGT vs patients with T2D classification classes. For the supervised UMAP embedding, the parameters were set to: n_components=2, verbose=True, metric=‘cosine’, n_epochs=1000, min_dist=0.5, n_neighbors=50, random_state=1, target_weight=0.6 and their respective class label information. The imputation *via* diffusion is controlled by parameters such as the diffusion time (*t)*, the decay rate (*decay*), and the number of nearest neighbors to consider (*knn*). We set t=1, decay=50, and *knn*=3. This choice of parameters was to preserve the structure as much as possible avoiding over-smoothing of the abundances to other classes. After using the mb-PHENIX algorithm, the GM profile values (ASV tables) were independently normalized (Log2) but only in the necessary methods, such as Logistic Regression, Naive Bayes, and MLP.

We investigated alpha diversity indicators for the three different groups of normoglycemic, prediabetic, and diabetic individuals using the Shannon, Simpson, and InvSimpson index. Despite, we could not find a connection between the presence of T2D and microbial alpha diversity (**Supplementary Figure 4**). These results are in line with previous studies, which has been unable a clear relationship between microbial diversity and T2D (14, 22, 44). In addition, we performed a beta diversity analysis, but the results did not show any clear clustering patterns that could consistently distinguish between the groups of people with normoglycemia, prediabetes, and T2D (**Supplementary Figure 5**). These results highlight the complexity of the relationship between microbial diversity and T2D status.

### 4.3 Machine learning methods

Three comparisons were performed to assay the classifications: classification 1 (C1) compared patients with NGT versus patients with prediabetes; classification 2 (C2) compared patients with NGT versus patients with T2D; classification 3 (C3) we made a multi-class classification to predict individuals with NGT, prediabetes, and T2D. We developed a base pipeline for each classification to train and evaluate each model. The linear ML methods used included: Binary Logistic Regression and Naive Bayes. The nonlinear ML methods used included: Decision Tree, Random Forest, and XG Boost. Additionally, MLP with a Multilayer Perceptron (MLP) architecture was used in each classification.

After the preprocessing step, we randomly split the database into a training set (80%) and a test set (20%). Subsequently, each model was individually trained using the training subset (80%) with the different ML algorithms. And at the end, the model’s performance was evaluated using the data from the test set (20%). This evaluation was performed using the values for accuracy and AUC-ROC values (**Tables 1**, **2**). In the case of the multiclass classification, we evaluated it using the Cohen Kappa score (**Table 3**).

**Table 1:** Classification 1 (C-1) performance (Patients with NGT vs. Patients with prediabetes).

ML algorithms	Accuracy	AUC	Mean Accuracy (SD, CV=10)	Mean AUC (SD, CV=10)
Logistic Regression	0.94	0.95	0.9 (0.05)	0.94 (0.04)
Naive Bayes	0.78	0.78	0.76 (0.07)	0.87 (0.05)
Decision Tree	0.81	0.81	0.83 (0.08)	0.82 (0.08)
Random Forest	0.96	0.96	0.98 (0.02)	0.99 (0.01)
XGBoost	0.83	0.83	0.88 (0.04)	0.96 (0.01)
Multilayer Perceptron (MLP)	0.94	0.94	0.94 (0.02)	0.94 (0.03)

We compared the performance of six ML algorithms using the Precision and area under the receiver operating characteristic (ROC) curve (AUC)values. To obtain the standard deviation (SD) in our results, we use the stratified cross-validation (CV) technique (*K Fold* = 10).

**Table 2:** Classification 2 (C-2) performance (Patients with NGT vs. Patients with T2D).

ML algorithms	Accuracy	AUC	Mean Accuracy (SD, CV=10)	Mean AUC (SD, CV=10)
Logistic Regression	1	1	0.94 (0.04)	0.94 (0.06)
Naive Bayes	0.87	0.84	0.85 (0.04)	0.92 (0.04)
Decision Tree	0.86	0.83	0.84 (0.08)	0.75 (0.16)
Random Forest	0.89	0.98	0.96 (0.03)	0.99 (0.02)
XGBoost	0.92	0.96	0.91 (0.04)	0.98 (0.02)
Multilayer Perceptron (MLP)	1	1	0.91 (0.07)	0.93 (0.07)

We compared the performance of six ML algorithms using the Precision and area under the receiver operating characteristic (ROC) curve (AUC) values. We use the stratified cross-validation (CV) technique to obtain our results’ standard deviation (SD) (*K Fold* = 10).

**Table 3:** Classification 3 performance (C-3) (Patients with NGT vs. Patients with prediabetes vs. Patients with T2D).

ML algorithms	Accuracy	Cohen Kappa	Mean Accuracy (SD, CV=10)	Mean Cohen Kappa (SD, CV=10)
Logistic Regression	0.77	0.61	0.76 (0.06)	0.65 (0.11)
Naive Bayes	0.71	0.5	0.68 (0.07)	0.49 (0.12)
Decision Tree	0.6	0.29	0.65 (0.08)	0.35 (0.17)
Random Forest	0.98	0.63	0.95 (0.03)	0.9 (0.03)
XGBoost	0.92	0.87	0.96 (0.02)	0.93 (0.05)
Multilayer Perceptron (MLP)	0.93	0.87	0.9 (0.06)	0.88 (0.07)

We compared the performance of six ML algorithms using the Precision and Cohen Kappa score values. We use the stratified cross-validation (CV) technique to obtain our results’ median and standard deviation (SD) (*K Fold* = 10).

We also calculated their respective median value and SD using the stratified cross-validation technique (K Fold = 10) (**Figure 1**) for the performance metrics, including accuracy, AUC-ROC, and Cohen Kappa score. This comparison allowed us to select the model with the best predictive performance. Finally, an interpretive analysis of the best model for each classification was performed. Our study found Random Forest and XGboost as the best model’s performance; for this, we used TreeExplainer (45). It should be highlighted that the results of the SHAP values for each fold have been condensed into one plot for each classification task, as shown in **Supplementary Figure 3**.

Overall, this *post-hoc* analysis used the SHAP values to highlight the most important bacterial genera for correctly classifying healthy individuals or individuals with diabetes or at high risk of developing diabetes.

## Data availability statement

The original contributions presented in the study are included in the article/**Supplementary Material**. Further inquiries can be directed to the corresponding author.

## Ethics statement

The studies involving human participants were reviewed and approved by Research Council of the University of Guanajuato. The patients/participants provided their written informed consent to participate in this study.

## Author contributions

DN-R and OR-A conceived and designed the study. DN-R executed the experiments, analyzed the data, performed statistical tests, and drafted/revised the manuscript. YM-L, DE-H, DG-V, JS-C, AV-J, and CP-M analyzed the data, performed statistical tests, and contributed to the research design. All authors contributed to the writing of the manuscript and approved the final version.
