# Supplementary material for: Dysbiosis signatures of gut microbiota and the progression of type 2 diabetes: a machine learning approach in a Mexican cohort
Source: Front Endocrinol (Lausanne). 2023 Jun 27;14:1170459. doi: 10.3389/fendo.2023.1170459 (PMC10333697; doi:10.3389/fendo.2023.1170459)
Supplement: Supplementary file 1 [file DataSheet_1.docx]

Supplementary Material

Dysbiosis signatures of gut microbiota and the progression of type 2 diabetes: A machine learning approach in a Mexican cohort.

Neri-Rosario Daniel^1,2^, Martínez-López Yoscelina Estrella^1^, Esquivel-Hernández Diego A.^1^, Sánchez-Castañeda Jean Paul^1,2^, Padron-Manrique Cristian^1,4^, Vázquez-Jiménez Aarón^1^, Giron-Villalobos David^1,2^, Resendis-Antonio Osbaldo^1,3,5^*

^1^Human Systems Biology Laboratory. Instituto Nacional de Medicina Genómica (INMEGEN). México City, México.

^2^Programa de Maestría y Doctorado en Ciencias Bioquímicas, Universidad Nacional Autónoma de México (UNAM). Ciudad de México, México.^3^Coordinación de la Investigación Científica – Red de Apoyo a la Investigación, Universidad Nacional Autónoma de México (UNAM). Ciudad de México, México.

^4^Programa de Doctorado en Ciencias Biomédicas, Universidad Nacional Autónoma de México (UNAM). Ciudad de México, México.

^5^Centro de Ciencias de la Complejidad, Universidad Nacional Autónoma de México (UNAM). Ciudad de México, México.

*** Correspondence:**Corresponding Author
**oresendis@inmegen.gob.mx**

# 1. Supplementary Figures and Tables

| **ML method** | **Definition** | **Linearity** |
| --- | --- | --- |
| Logistic regression | Is a process of modeling the probability of a discrete outcome given an input variable. | Lineal. [(1)](https://paperpile.com/c/QPNQys/lOKK) |
| Naive Bayes | Calculates a class's probability given a set of feature values. Assume independence between attributes. | Lineal. [(1)](https://paperpile.com/c/QPNQys/lOKK) |
| Decision-Tree | Classify according to a series of questions and conditions. | Non-linear. [(2)](https://paperpile.com/c/QPNQys/RZ6V) |
| Random Forest | Tree-based ensemble learning method using a set of uncorrelated decision trees depending upon several randomly selected variables. | Non-linear. [(3)](https://paperpile.com/c/QPNQys/JInu) |
| XGBoost | Tree-based ensemble learning method using a set of decision trees is created by calculating the error of the previous model until the highest prediction is found. | Non-linear. [(4)](https://paperpile.com/c/QPNQys/7Sv8) |
| Multiple layer perceptron  (MLP) | ML technique to extract and transform information using multiple layers of neural networks. These layers receive information from previous layers and are progressively refined. These layers are trained using algorithms that minimize errors and improve prediction. | Non-linear [(5)](https://paperpile.com/c/QPNQys/K9EF) |

## Supplementary table 1. Definitions and characteristics of the ML methods used in this article.

| **Term** | **Description** |
| --- | --- |
| **Cohen's Kappa:** | **​​A statistical measure of inter-rater agreement for categorical items. It ranges from -1 to 1, with values closer to 1 indicating a higher level of agreement between raters.** |
| **AUC:** | **A measure that assesses how well a model can distinguish between two groups (e.g., healthy vs. diseased) by measuring the model's ability to correctly identify positive and negative cases. Ranges are from 0.5 (random guessing) to 1 (perfect classification).** |
| **Supervised ML:** | **A type of machine learning where the algorithm is trained on labeled data to make predictions based on input features.** |
| **ML explainability analysis:** | **A process to identify which features or variables are most important for a machine learning model's predictions in order to understand the reasoning behind the model's decisions.** |
| **SHAP value:** | **An acronym for "SHapley Additive exPlanations." It is a method used to explain the output of a machine learning model by assigning importance scores to each input feature.** |

1.2 Supplementary Table 2: Side-note box: Terminology.

##
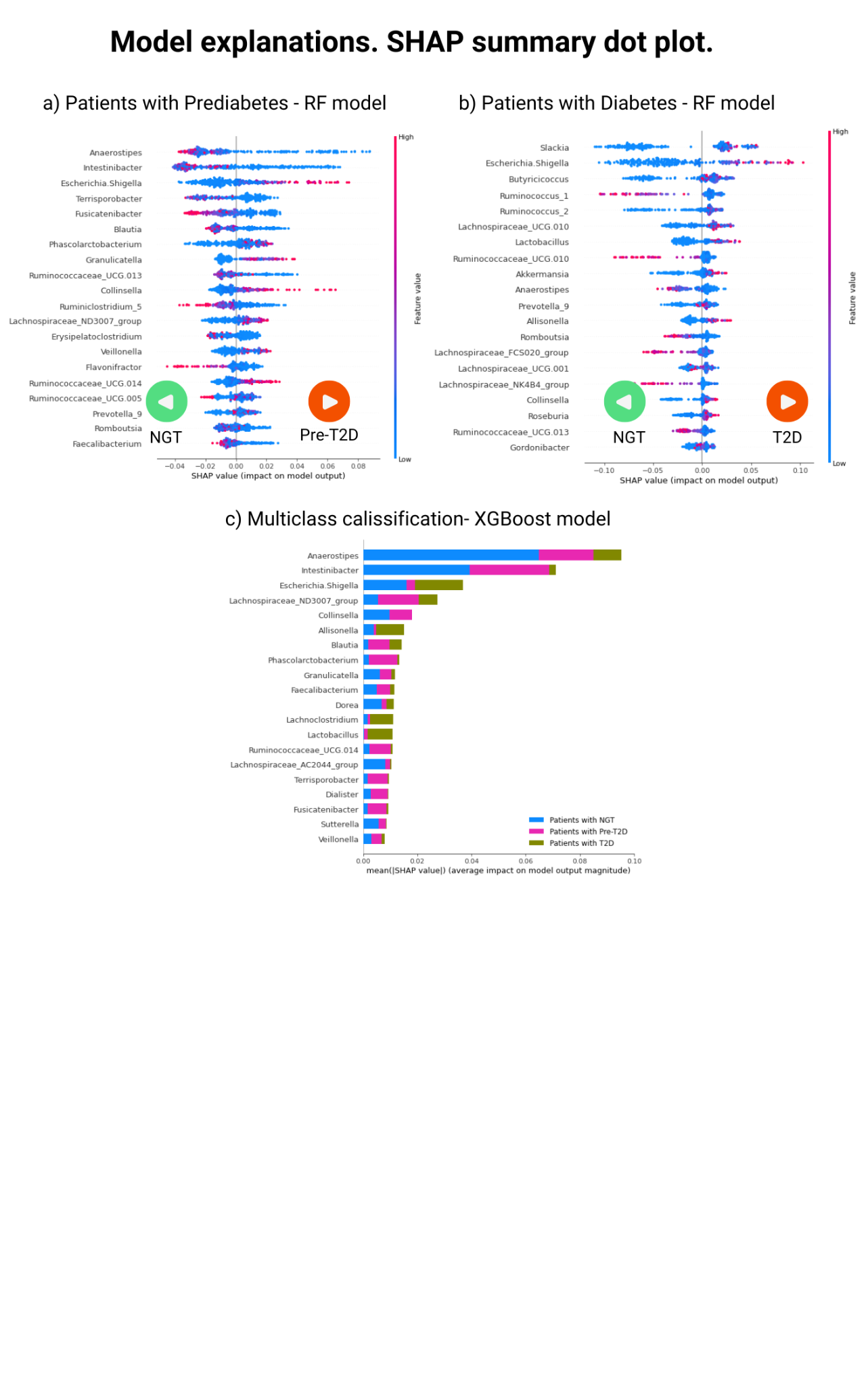


## Supplementary figure 1. Performance comparison between ML models without mb-Phenix. We compared six ML algorithms in three classifications a) Individuals with NGT vs. Patients with prediabetes, b) Patients with NGT vs. Patients with T2D, and c) Patients with NGT vs. Patients with prediabetes vs. Patients with T2D. We plot a standard error bar of AUC-ROC median values for a visual comparison performance between the models in each classification. In the case of multi-class classification (part C), we evaluated it using Cohen Kappa Score. The base pipeline workflow for all models followed several steps. First, the database was randomly split into a training set (80%) and a test set (20%). Next, the gut microbiome profile values (ASV abundance tables) were normalized (log2) in both sets, and the SMOTE algorithm was applied to address any imbalance in the data. This step was only performed in methods that require normalization, such as logistic regression, naive Bayes, and artificial neural networks. Afterward, each model was individually trained using all the data in the training set. Finally, the accuracy of each model was evaluated using the data from the test set, with the accuracy and AUC-ROC metrics used for binary classification and the accuracy metric and Cohen's Kappa index used for multiclass classification (SM3 simulation). We evaluated using a stratified cross-validation technique (K fold = 10).

##
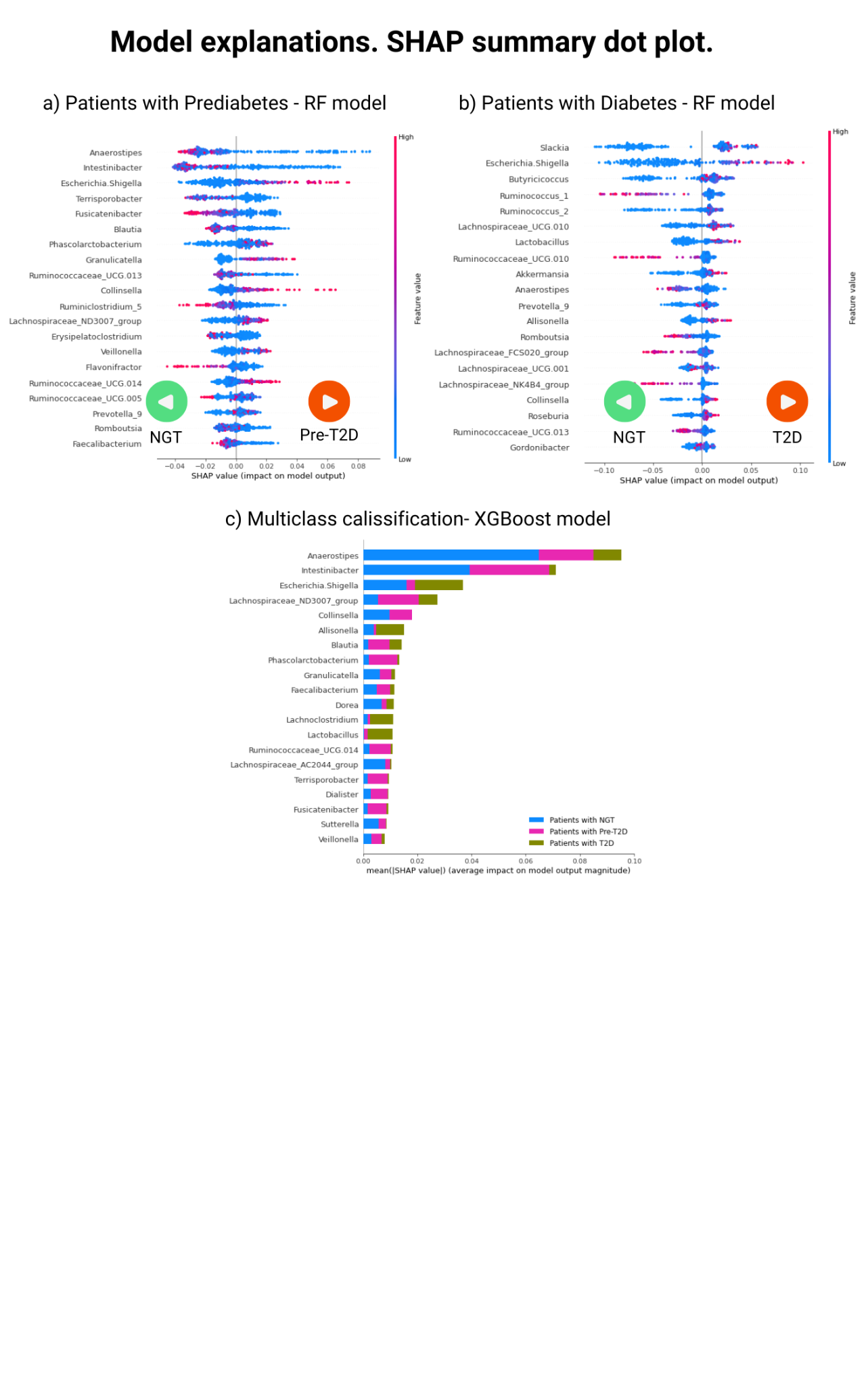


## Supplementary figure 2. Model evaluation using summary SHAP plot without mb-PHENIX. We analyze the three comparisons. a) Patients with NGT (n= 213) versus patients with prediabetes (n= 47). b) Patients with NGT (n= 213) versus patients with T2D (n= 47). c) Multi-class classification: Patients with NGT vs. patients with prediabetes (n=150) vs. patients with T2D (n= 47). RF (Random Forest), pre-T2D (prediabetes).

##
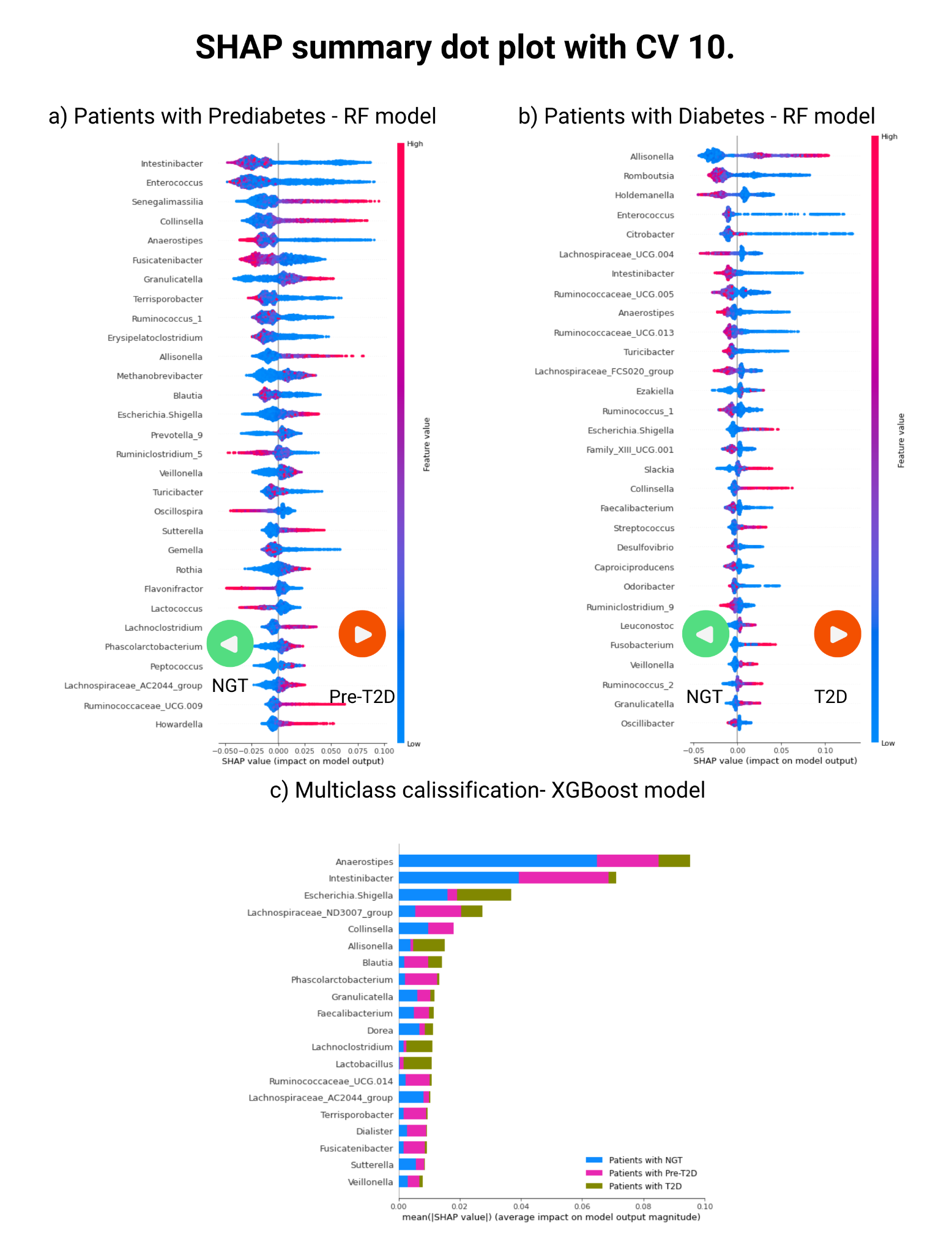


## Supplementary figure 3. We condensed the results of the SHAP values for each fold into one plot for each of the three comparisons. a) Patients with NGT (n= 213) versus patients with prediabetes (n= 47). b) Patients with NGT (n= 213) versus patients with T2D (n= 47). c) Multi-class classification: Patients with NGT vs. patients with prediabetes (n=150) vs. patients with T2D (n= 47). RF (Random Forest), pre-T2D (prediabetes).


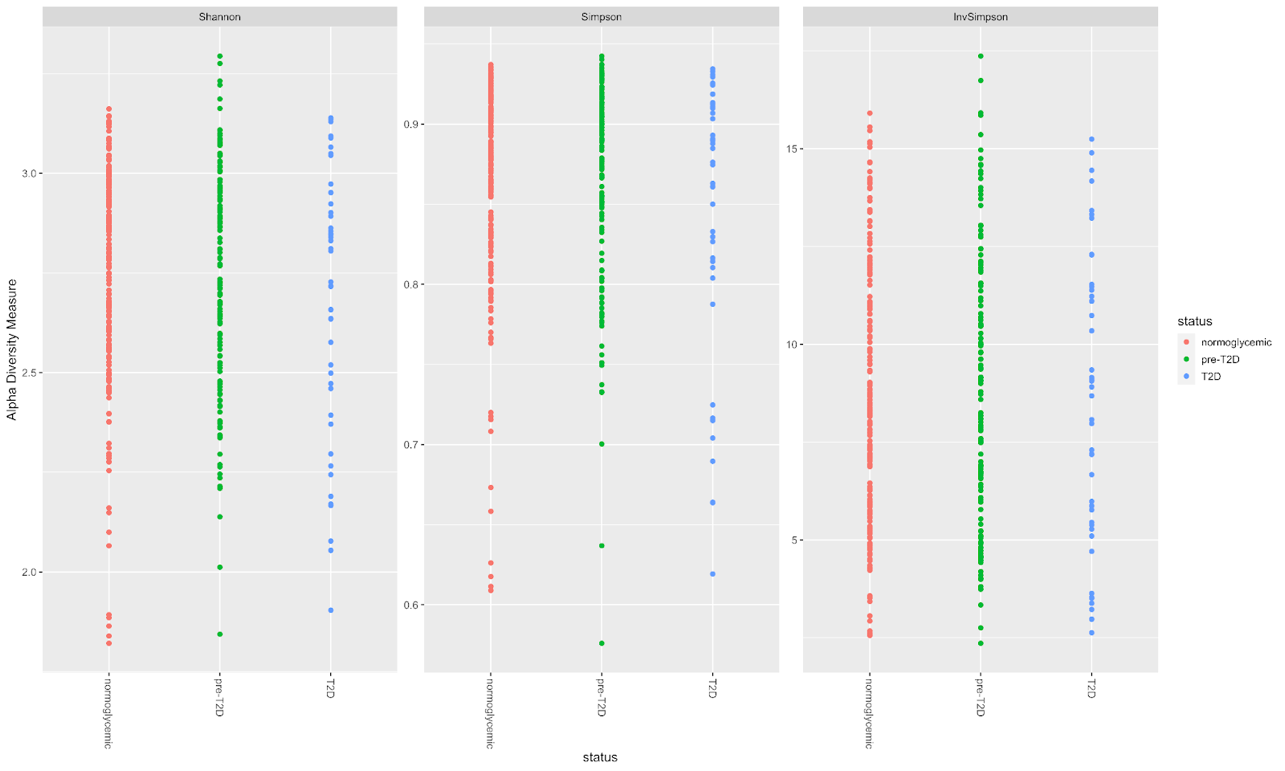


1.5 Supplementary Figure 4. Alpha diversity (Shannon, Simpson, InvSimpson).


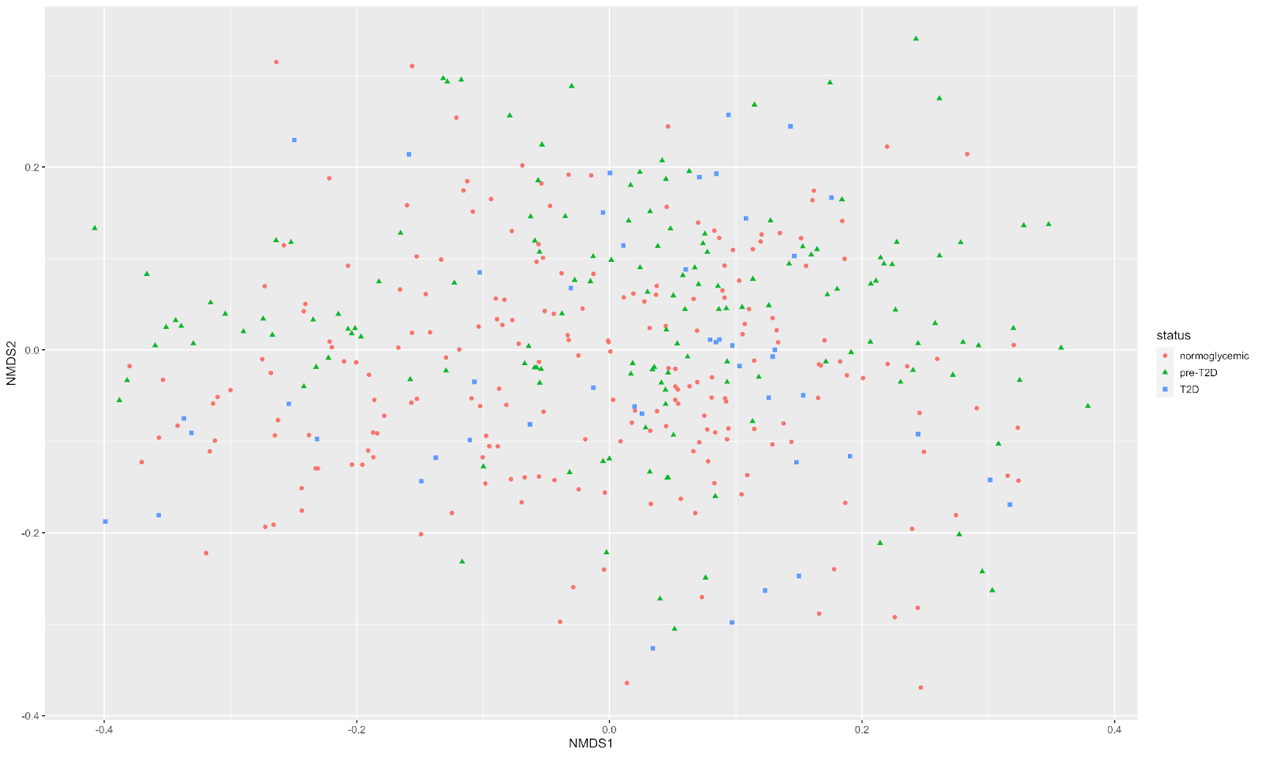


1.6 Supplementary Figure 5 Beta analysis diversity. It shows that despite the microbial variety between the groups, we are unable to discern a distinctive group pattern.


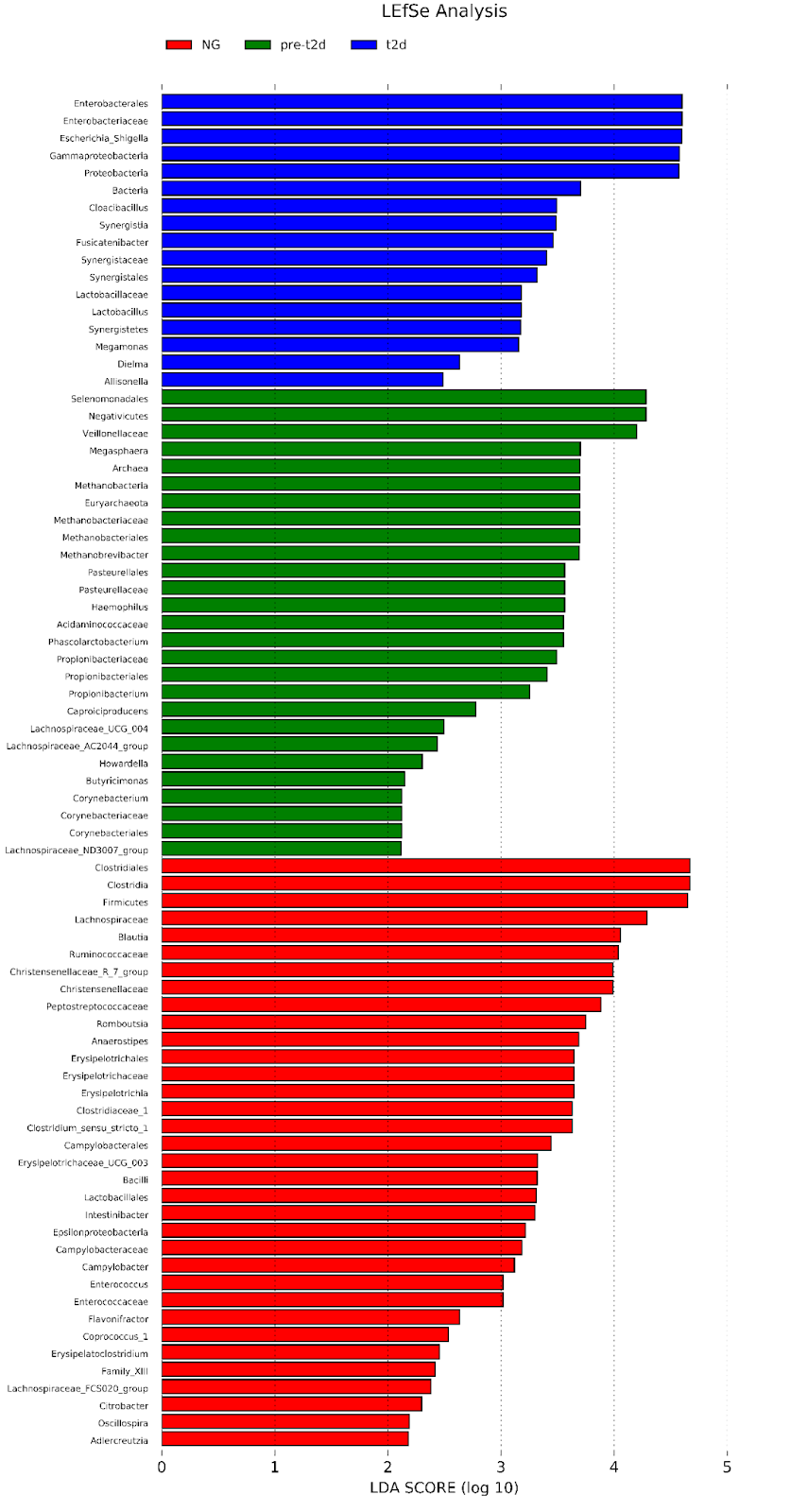


1.7 Supplementary Figure 6. Barplot of the linear discriminant analysis (LDA) of the GM between normoglycemic, pre-T2D, and T2D at the genus level (6).  The bars are marked with significant taxa that help to distinguish the groups. The normoglycemic (blue), pre-T2D (green), and T2D (red) study groups are represented by the different colored bars.


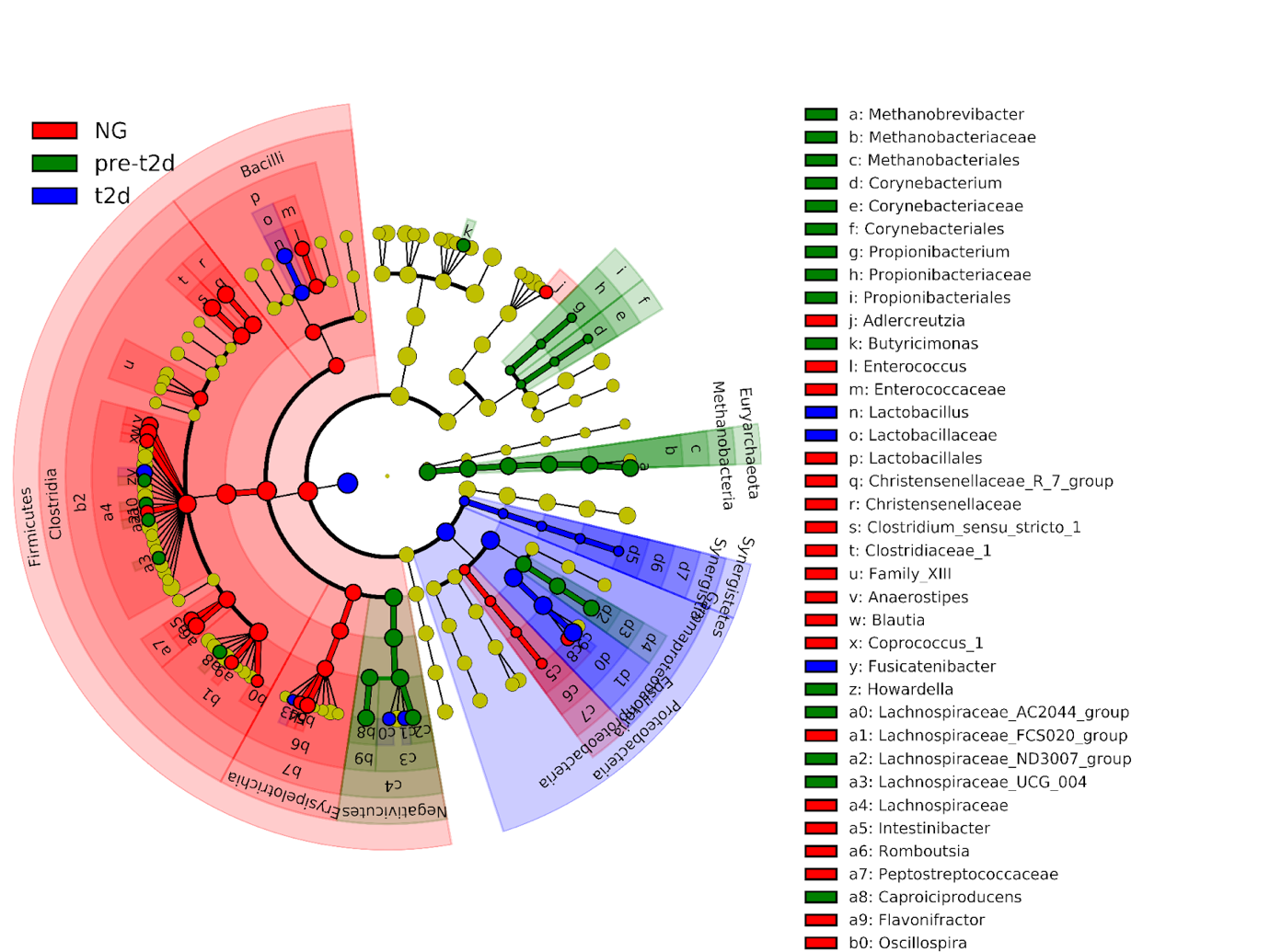


1.7 Supplementary Figure 7. Cladogram of Linear discriminant analysis Effect Size (LEfSe) analysis (6). Each node represents a distinct taxonomic group, and the size of the node denotes the magnitude of the influence. The nodes' colors—normoglycemic (blue), pre-T2D (green), and T2D (red)—reflect the various study groups.

# 2. REFERENCES

1. [Zhou YH, Gallins P. A Review and Tutorial of Machine Learning Methods for Microbiome Host Trait Prediction. Front Genet. 2019 Jun 25;10:579.](http://paperpile.com/b/QPNQys/lOKK)

2. [Quinlan JR. Induction of decision trees. Mach Learn. 1986 Mar 1;1(1):81–106.](http://paperpile.com/b/QPNQys/RZ6V)

3. [Breiman L. Random Forests. Mach Learn. 2001 Oct 1;45(1):5–32.](http://paperpile.com/b/QPNQys/JInu)

4. [Chen T, Guestrin C. XGBoost: A Scalable Tree Boosting System. In: Proceedings of the 22nd ACM SIGKDD International Conference on Knowledge Discovery and Data Mining. New York, NY, USA: Association for Computing Machinery; 2016. p. 785–94. (KDD ’16).](http://paperpile.com/b/QPNQys/7Sv8)

5. [Sarker IH. Deep Learning: A Comprehensive Overview on Techniques, Taxonomy, Applications and Research Directions. SN Comput Sci. 2021 Aug 18;2(6):420.](http://paperpile.com/b/QPNQys/K9EF)

6. Segata N, Izard J, Waldron L, Gevers D, Miropolsky L, Garrett WS, et al. Metagenomic biomarker discovery and explanation. Genome Biol. 2011 Jun 24;12(6):R60.
